# Veritable antiviral capacity of natural killer cells in chronic HBV infection: an argument for an earlier anti-virus treatment

**DOI:** 10.1186/s12967-017-1318-1

**Published:** 2017-10-31

**Authors:** Xiaoyan Li, Liang Zhou, Lin Gu, Yurong Gu, Lubiao Chen, Yifan Lian, Yuehua Huang

**Affiliations:** 1 Department of Infectious Diseases, The Third Affiliated Hospital of Sun Yat-sen University, No. 600 Tianhe Road, Guangzhou, 510630 China; 2 Guangdong Provincial Key Laboratory of Liver Disease Research, The Third Affiliated Hospital of Sun Yat-sen University, No. 600 Tianhe Road, Guangzhou, 510630 China

**Keywords:** Hepatitis B virus, Natural killer cells, Immune tolerant, Anti-viral treatment

## Abstract

**Background:**

There is limited information on innate immunity, especially natural killer (NK) cell function, in different chronic hepatitis B (CHB) stages. Therefore, we examined whether the clinical staging strategy accurately reflects veritable NK cell immunity.

**Methods:**

A total of 237 eligible CHB patients and 22 healthy controls were enrolled in our study. Demographic and clinical data were collected, and the CHB phases (immune active-IA, immune tolerant phase-IT, inactive CHB-IC, and grey zone-GZ) were classified according to the latest American Association for the Study of Liver Disease guidelines. Peripheral blood mononuclear cells from patients and healthy controls were tested for NK cell frequency, phenotype and function using flow cytometry.

**Results:**

A significant decrease in activating receptor NKp44 and NKp46 expression and significant increase of exhaustion molecule Tim-3 expression were observed in NK cells from CHB patients. Reduced cytokine secretion and preserved or elevated cytotoxic function were also observed. Patients in the IT group exhibited comparable cytokine secretion and cytolytic capacity as age-matched IA patients. NK cell anti-viral functions were preserved in GZ patients. Some of the NK cell function in patients who were excluded from treatment by the current treatment guidelines was less compromised than patients who qualified for treatment.

**Conclusion:**

Our findings provide evidence of veritable NK cell immunity during different natural history phases in treatment-naïve patients with chronic HBV Infection. Chronic HBV infection hindered NK cell function in CHB patients. However, the presumed IT and GZ statuses of CHB patients based on the clinical parameters may not accurately reflect the inner immune status of these patients and should be reconsidered. Some patients excluded from treatment by the current treatment guidelines may be able to be selected as candidates for treatment.

**Electronic supplementary material:**

The online version of this article (doi:10.1186/s12967-017-1318-1) contains supplementary material, which is available to authorized users.

## Background

Hepatitis B virus (HBV) is one of the most dangerous and prevalent infectious agents and affects approximately 240 million people worldwide [1]. Chronic HBV infection leads to liver diseases, such as cirrhosis, hepatocellular carcinoma, and liver failure. The clinical stages of CHB are generally classified as the immune tolerant phase (IT), immune active phase (IA) and inactive CHB phase (IC) based on serum alanine aminotransferase (ALT), HBV DNA levels and HBeAg status [1, 2]. Some patients cannot be classified into the above stages and fall into the grey zone (GZ) [3]. Current guidelines recommend the presence of an active liver inflammation using histology or elevated ALT serum levels greater than twofold the ULN plus elevated HBV DNA above 2000 IU/mL (HBeAg negative) or 20,000 IU/mL (HBeAg positive) as criteria for initiating antiviral therapy [1]. Progression of chronic HBV infection results from ineffective attempts of the host immune response to eliminate the virus. However, the innate and adaptive immune responses against HBV in chronic hepatitis B (CHB) patients are compromised [4]. The exhausted HBV-specific T cell response is presumably the primary cause of HBV persistence, but functional deficiency of other components of the innate immune system, such as natural killer (NK) cells and dendritic cells (DC), are also involved [5].

NK cells are enriched in the liver (30%) compared to the blood (5–20%), and their percentage is further increased in viral hepatitis patients [6]. NK cells express a combination of activating and inhibitory receptors. NK cells are functionally triggered when the signalling of activating receptors overcomes the signalling of inhibitory receptors. Reports on receptor expression during chronic persistent HBV infection are inconsistent [7–11]. NK cells play a key role in antiviral immunity by identifying and killing infected target cells and secreting inflammatory cytokines, such as interferon-γ (IFN-γ) [12]. NK cells also regulate the adaptive immune response by interacting with APCs and T cells, which affects the development of HBV infection [7]. Individuals who clear HBV during acute HBV infection exhibit good NK cell responses against HBV and express more activating receptors and fewer inhibitory receptors [13]. By contrast, NK cell function is disrupted in patients with prolonged HBV infection, which weakens the eradication of HBV from the liver [14].

Previous studies observed decreased cytokine secretion from NK cells in CHB patients [12], but few studies have focused on NK cell features during the different phases of CHB, especially phases defined according to the new upper limit of normal (ULN) of ALT (30 U/L for males and 19 U/L for females) according to the latest American Association for the Study of Liver Disease guidelines. Data comparing veritable NK cell features in patients who are recommended to initiate antiviral therapy (CA) with patients who are excluded from treatment according to the guidelines (CAN) are also lacking. The concept of IT has also been challenged [12], and it is unclear how virus–host relationships, age, and tolerance are related during this stage [15].

This study analysed the frequency, phenotype, and function of NK cells in a large cohort of well-characterized CHB patients in different clinical stages. We report an inhibitory phenotype and impaired function of NK cells in CHB patients as a whole and demonstrated the functional features of NK cells in CHB patients in different clinical phases. Notably, we found a partially preserved function of NK cells in patients who were excluded for treatment according to the guidelines (CAN) and patients in IT and GZ patients. The veritable NK cell features in the different CHB patient groups may provide more information for CHB treatment guidelines.

## Patients and methods

### Patients

Patients with HBV infection for at least 6 months from the outpatient hepatology clinic of the Third Affiliated Hospital of Sun Yat-Sen University (Guangzhou, China) were enrolled for this study from July 2015 to January 2017. Patients who received antiviral treatment (IFN-α or nucleoside analogues) previously, patients with human immunodeficiency virus (HIV), hepatitis C virus, or hepatitis D virus co-infection, and patients with end-stage liver insufficiency, autoimmune disorders, immunosuppressive treatment, cirrhosis, or malignancies were excluded. The study was performed according to The Code of Ethics of the World Medical Association, and informed consent was obtained from all participants. The Institute Review Board of the Third Affiliated Hospital of Sun Yat-Sen University approved the study protocol.

A total of 237 eligible CHB patients were included in this study for analysis. The classification of CHB patients in this study was based exclusively on serological and biochemical parameters in accordance with published international treatment guidelines (Table 1) [1]. Twenty-two healthy controls (HC) were included in this study. Table 2 lists information on the demographics (age range and gender distribution), body mass index (BMI), HBV markers [HBeAg, HBV DNA, HBV surface antigen (HBsAg), anti-HBeAg], hepatic panel [albumin (ALB), ALT, total bilirubin (TBIL)], HBV genotypes, and liver stiffness measurements (Fibroscan value).

**Table 1:** Classification criteria

Classification	ALT	HBV DNA	HBeAg
Disease phases classification			
Immune active (IA)	Elevated	> 20,000 IU/mL	Positive
		> 2000 IU/mL	Negative
Inactive CHB (IC)	Normal	Low HBV DNA level	Negative
Immune tolerance (IT)	Normal	> 1 million IU/mL	Positive
Grey zone (GZ)	Not classified as IC, IT or IA		
Treatment classification			
CHB patients who are recommended to initiate antiviral therapy (CA)	> 2 ULN	> 20,000 IU/mL	Positive
		> 2000 IU/mL	Negative
CHB patients who are not strongly recommended to be given antiviral therapy (CAN)	Patients did not meet CA criteria		

Upper limit of normal (ULN) of ALT: 30 U/L for males and 19 U/L for females

**Table 2:** Clinical-virological characteristics of patients included in the study

Characteristics	CHB (n = 237)
Age, years	29 (25, 35)
Gender, n (%)	
Female	69 (29.1)
Male	168 (70.9)
Body mass index	21.1 (19.3, 23.2)
HBV DNA, log 10 IU/mL	4.72 (3.14, 8.23)
HBV genotype, n (%)	
B	120 (50.6)
C	50 (21.1)
Other	67 (28.3)
HBeAg status, n (%)	
HBeAg positive	128 (54.0)
HBeAg negative	108 (45.6)
Missing	1 (0.004)
HBsAg, IU/mL	3499 (987.2, 26,482.5)
Smoker, n (%)	
No	25 (10.5)
Yes	212 (89.5)
Vertical transmission, n (%)	
No	177 (74.7)
Yes	31 (13.1)
Missing	29 (12.2)
ALT, U/L	32.0 (23.0, 54.0)
TBIL, mg/dL	13.10 (10.30, 17.07)
ALB, g/L	46.1 (44.1, 47.7)
FibroScan value, kPa	5.0 (4.3, 6.1)

Continuous variables were showed median (25, 75%)

### Flow cytometry analysis

Peripheral blood mononuclear cells (PBMCs) were isolated from the fresh blood of patients using Ficoll density gradients as described previously [16]. Isolated PBMCs were stained for surface markers, fixed, permeabilised with IntraPreReagent (Beckman Coulter, Fullerton, CA) and further stained with antibodies directed against intracellular markers. Leukocytes were stimulated with Leukocyte Activation Cocktail (BD Bioscience, USA) at 37 °C for 4 h prior to intracellular staining using the manufacturer’s staining protocol. Anti-human mAbs against CD3-PE-CF594, CD56-FITC, NKG2D-PE, NKp46-PE-CY7, NKp-30-APC, NKp44-PE, NKG2A-APC, CD69-PE-CY7, PD1-Pacific blue, Tim-3-APC, perforin-APC, Granzyme B-BV421, IFN-γ-PE, and TNF-α-PE with corresponding isotype-matched controls were purchased from BD Biosciences (San Jose, CA, USA). Data were acquired on a Gallios instrument (Beckman Coulter, Brea, CA, USA) and analysed using FlowJo software (Flow jo, LCC, USA).

### Clinical and serological parameters

Serum was tested for HBsAg, anti-HBsAg, HBeAg and anti-HBeAg using commercial kits (Abbott Laboratory, North Chicago, IL) upon recruitment to the study. The HBV genotype was determined using direct sequencing. Quantitative HBsAg was measured using Elecsys HBsAg II Quant reagent kits (Roche Diagnostics, Indianapolis, IN) according to the manufacturer’s instructions. Serum HBV DNA level was measured using the Roche COBAS Ampliprep/COBAS Taqman HBV test v2.0 (dynamic range from 20 to 1.7E+08 IU/mL, Roche Molecular Diagnostics, Branchburg, NJ). Fibrosis levels were defined using liver stiffness measurements (Fibroscan, Echosens, Paris, France).

### Statistical analysis

We compared patient groups using the Mann–Whitney U test and one-way ANOVA for continuous variables and the Chi square (χ^2^) test for categorical variables. Data are expressed as medians. We examined the association between two continuous variables using the Pearson correlation coefficient (r) and a linear regression model. All statistical tests were performed using R software version 3.2.2. A p < 0.05 was considered statistically significant. p values in figures are illustrated as *p < 0.05; **p < 0.01; and ***p < 0.001.

## Results

### Frequency of circulating NK cells in treatment-naïve CHB patients

**Fig. 1:**
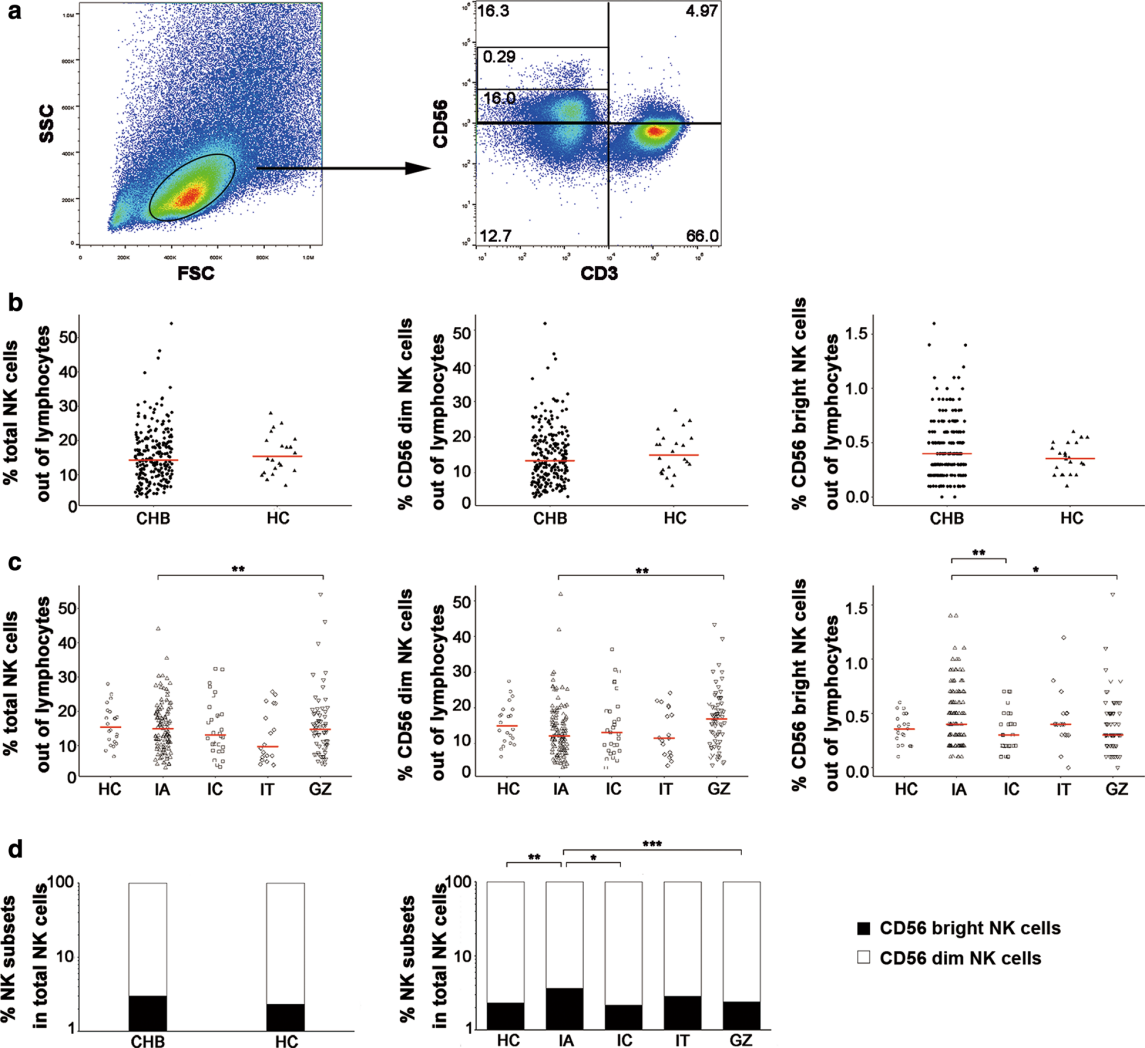
NK cell frequency and subset distribution in CHB patients. **a** NK cells were identified within PBMCs after gating of lymphocytes. CD56+CD3− NK cells were subsequently gated into CD56 bright and CD56 dim subsets by CD56 expression intensity. Percentages of total NK, CD56 dim and CD56 bright NK cells in total lymphocytes are shown. The data shown are representative of at least ten individuals from more than three independent experiments. **b** The frequency of total NK cells and their two subsets are shown for CHB patients (CHB) and healthy controls (HC). **c** The frequency of total NK cells and their two subsets in patients are shown for healthy controls (HC) and subgroups of the different clinical phases of CHB. **d** Distribution of CD56 dim and CD56 bright NK subsets, which are depicted as the frequency of CD56+CD3− NK cells. *CHB* chronic hepatitis B, *HC* healthy control, *IA* immune active, *IT* immune tolerance, *IC* inactive CHB, and *GZ* grey zone

NK cells represent approximately 5–20% of the total lymphocyte population and 30% of intrahepatic lymphocytes, which makes these cells an important component of the innate immune system. We detected the percentage of NK cells and their subsets in total lymphocytes from CHB patients in four clinical phases (IA, IC, IT, and GZ) and healthy controls to investigate whether the distributions of total NK cells and their subsets change within distinct disease phases in treatment-naïve CHB patients. Tables 2 and 3 show the characteristics of the patients studied. No significant differences in the percentages of total NK cells or their subsets were observed between CHB patients and healthy controls (Fig. 1b). However, the frequency of total NK and CD56 dim NK cells in GZ patients was significantly higher than IA patients (Fig. 1c). The percentage of CD56 bright NK cells in IC and GZ patients was significantly reduced compared to IA patients (Fig. 1c). There was no significant difference in the distribution of NK cell subsets between CHB patients and healthy controls, but there were more CD56 bright NK cells in IA patients than IC, GZ and HC patients (Fig. 1d). This result suggests a shift in the NK cell compartment towards more CD56 bright NK cells in IA patients.

**Table 3:** Clinical-virological characteristics of patients in different clinical stages

Characteristics	IA (n=121)	IC (n=30)	IT (n=18)	GZ (n=68)
Age, years	29 (25, 34)	32 (27.75, 37)	25.5 (24, 26)	31 (26, 39)
Gender, n (%)				
Female	43 (35.5)	5 (16.7)	6 (33.3)	15 (22.1)
Male	78 (64.5.8)	25 (83.3)	12 (66.7)	53 (77.9)
Body mass index	20.8 (19.1, 22.6)	22.2 (21.0, 23.6)	20.8 (18.7, 22.5)	21.4 (19.6, 23.4)
HBV DNA, log 10 IU/mL	7.7 (5.0, 8.2)	2.4 (1.7, 3.0)	8.2 (8.2, 8.2)	3.2 (1.9, 4.2)
HBV genotype, n (%)				
B	73 (60.3)	11 (36.7)	12 (66.7)	24 (35.3)
C	31 (25.6)	4 (13.3)	3 (16.7)	12 (17.6)
Other	17 (14.1)	15 (50)	3 (16.7)	32 (47.1)
HBeAg status, n (%)				
HBeAg positive	79 (65.3)	3 (10)	18 (100)	8 (11.8)
HBeAg negative	42 (34.7)	27 (90)	0	59 (86.8)
Missing	0 (0)	0 (0)	0 (0)	1 (1.4)
HBsAg, IU/mL	7192 (1982, 45,333)	855 (82,1848)	40,369 (28,156, 52,000)	1571 (195, 3754)
Smoker, n (%)				
No	113 (93.4)	23 (76.7)	16 (88.9)	60 (88.2)
Yes	8 (6.6)	7 (23.3)	2 (11.1)	8 (11.8)
Vertical transmission, n (%)				
No	91 (75.2)	24 (80)	15 (83.3)	47 (69.1)
Yes	14 (11.6)	3 (10)	3 (16.7)	11 (16.2)
Missing	16 (13.2)	3 (10)	0 (0)	10 (14.7)
ALT, U/L	49 (34, 97.75)	17 (14, 25)	20 (16.5, 26.5)	28 (20.5, 33)
TBIL, mg/dL	13.7 (10.9, 18.7)	13.2 (11, 16.8)	14.8 (10.2,19.1)	11.4 (9.2, 14.5)
ALB, g/L	45.3 (43.35, 47.15)	47 (46, 49)	45.5 (44.0, 47.3)	46.9 (45.3, 48.1)
FibroScan value, kPa	5.5 (4.325, 6.475)	4.7 (4.225, 5.45)	4.85 (4.3, 5.4)	4.8 (4.4, 5.4)

Continuous variables were showed median (25, 75%)

### A panel of receptors on NK cells in treatment-naïve CHB patients

**Fig. 2:**
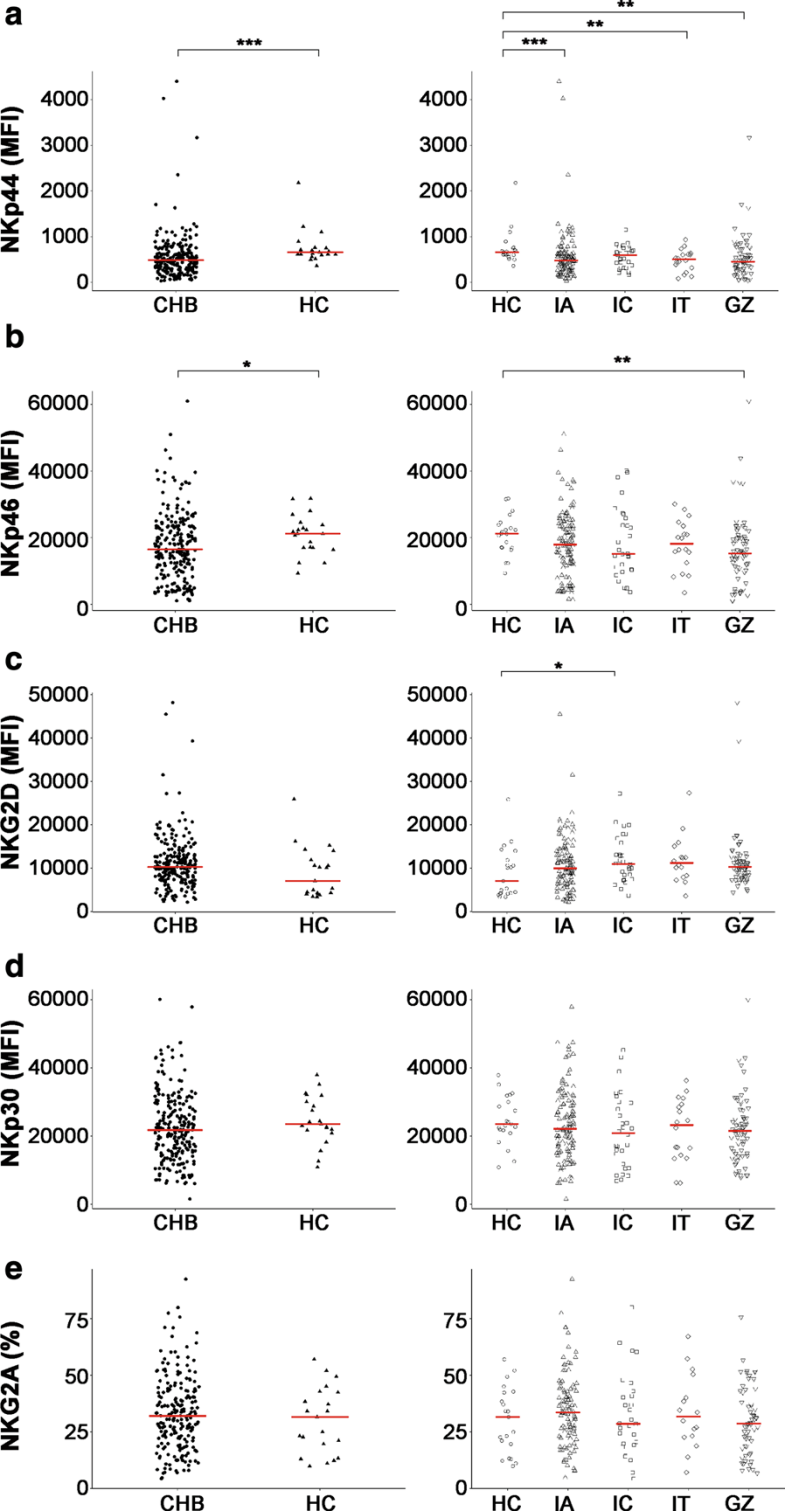
Receptor expression characteristics in treatment-naïve CHB patients. MFI for NKp44 (**a**), NKp46 (**b**), NKG2D (**c**), and NKp30 (**d**) on CD56+CD3− NK cells and the frequency for NKG2A^+^ cells (**e**) within CD56+CD3− NK cells in healthy controls (HC) and CHB patients (CHB)/CHB subgroups. Comparison between the HC group and total CHB group is shown on the left plots and comparisons between the HC group and different CHB subgroups that represent the different clinical phases are shown on the right plots. Horizontal bars represent the median value. *CHB* chronic hepatitis B, *HC* healthy control, *IA* immune active, *IT* immune tolerance, *IC* inactive CHB, and *GZ* grey zone

NK cell receptor (NKR) expression regulates NK cell function. Therefore, we investigated the expression of a panel of NKRs, including activating receptors NKp44, NKp46, NKG2D, and NKp30 and the inhibitory receptor NKG2A (Fig. 2a–e). Figure 2 and Additional file 1: Figure S1 show that the expression of activating receptors NKp44 and NKp46 in total NK cells and their subsets in the CHB cohort exhibited a decreasing trend compared to HC subjects. These differences were statistically significant, with the exception of NKp44 on CD56 bright NK cells. Varying degrees of decreased NKp44 expression were observed in CHB patients (Fig. 2a and Additional file 1: Figure S1A). The average level of NKp46 expression was lower in CHB patients than HC patients, but statistically significant differences were only observed in the total NK cell population and CD56 dim subset between the GZ and HC groups. There was also a statistically significant difference in the CD56 bright subset between the IC and HC groups (Fig. 2b and Additional file 1: Figure S1B). An up-regulation of NKG2D expression was observed in IC and GZ patients compared to the HC group (p = 0.0289; and p = 0.0501, respectively, Fig. 2c). A similar trend was observed in the CD56 dim and CD56 bright subsets, and the IC group exhibited significantly up-regulated NKG2D expression on CD56 bright NK cells. No other significant differences in activating receptor NKp30 or inhibitory receptor NKG2A expression were observed in the total NK cell populations of these groups (Fig. 2d, e).

### Functional profiles of NK cells in treatment-naïve CHB patients

**Fig. 3:**
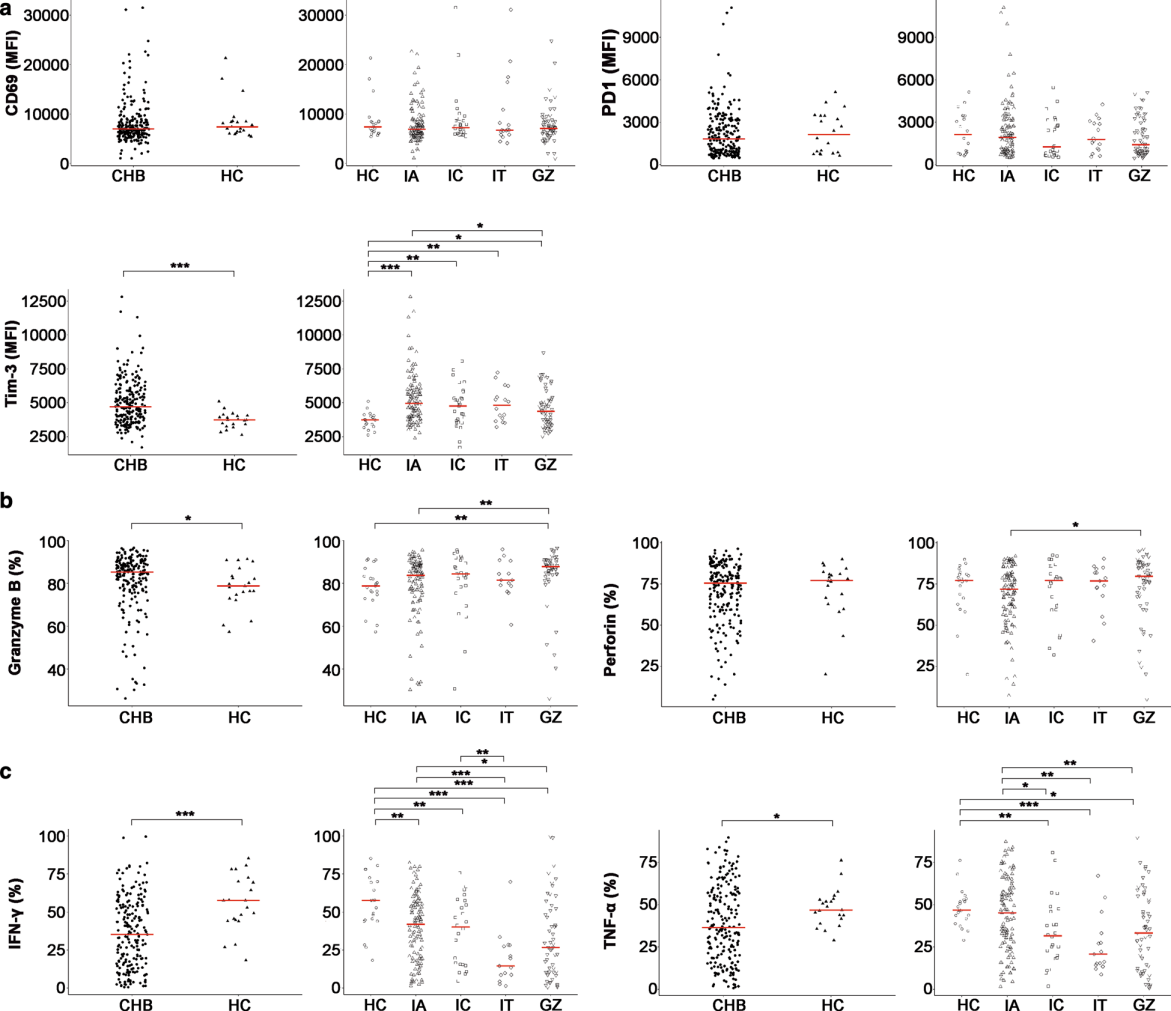
Chronic HBV infection affected the function of NK cells. MFI for CD69, PD1 and Tim-3 (**a**) on CD56+CD3− NK cells and the frequency of granzyme B^+^, perforin^+^ (**b**), IFN-γ^+^ and TNF-α^+^ (**c**) cells within CD56+CD3− NK cells in healthy controls (HC) and CHB patients (CHB)/CHB subgroups. Horizontal bars represent the median. *HBV* hepatitis B, *CHB* chronic hepatitis B, *HC* healthy control, *IA* immune active, *IT* immune tolerance, *IC* inactive CHB, and *GZ* grey zone

The innate immune responses during different clinical phases of CHB infection remain controversial [17]. Therefore, we analysed the activation status and cytotoxicity capability of NK cells and antiviral cytokine secretion by NK cells in CHB patients at different disease stages. The percentage of NK cells expressing the activation marker CD69 was not significantly different between patients in the four disease phases of CHB compared to the healthy controls (Fig. 3a). However, significantly higher CD69 expression was observed on CD56 bright NK cells in the IA group compared to the IC group (Additional file 1: Figure S2A). We also investigated the expression of two critical exhaustion molecules, PD1 and Tim-3 [18, 19]. PD1 expression in NK cells did not differ between the different clinical stages of CHB patients and healthy controls (Fig. 3a). However, Tim-3 expression in the total NK cell population was significantly higher in CHB patients and each stratified CHB subgroup compared to HC subjects. Significantly higher Tim-3 expression was also observed in the IA group compared to the GZ group (Fig. 3a).

We analysed cytotoxic capabilities using granzyme B and perforin levels. Our data demonstrated significantly more granzyme B^+^ NK cells (mean value, 85.30%) in CHB patients than HC subjects (mean value, 78.80%) (Fig. 3b). Granzyme B^+^ CD56 dim subsets were also more abundant in CHB patients than HC subjects (Additional file 1: Figure S2D). However, the production of perforin was comparable between groups (Fig. 3b). Total NK cells and the CD56 dim subset produced more granzyme B and perforin in the GZ group than the IA group and HC subjects. A higher level of granzyme B on total NK cells and the CD56 dim subset was also observed in the GZ group compared to the HC group (Fig. 3b and Additional file 1: Figure S2D, E).

Analysis of cytokine production by NK cells revealed that two major antiviral cytokines, IFN-γ and TNF-α [20, 21], decreased significantly in CHB patients compared to the HC group. Similar down-regulated cytokine production was observed in each CHB phase compared to the HC group, and this difference was statistically significant, with the exception of TNF-α in the IA patients (Fig. 3c). The IT group exhibited decreased cytokine secretion capability, with 14.60% IFN-γ^+^ and 20.80% TNF-α^+^ NK cells on average compared to 41.10% IFN-γ^+^ and 45.20% TNF-α^+^ NK cells in the IA group and 57.70% IFN-γ^+^ and 46.90% TNF-α^+^ NK cells in the HC group (Fig. 3c). The IA group possessed a great amount of IFN-γ^+^ NK cells and TNF-α^+^ NK cells than the IC and GZ groups (Fig. 3c). The changes in IFN-γ^+^ and TNF-α^+^ cells in two NK subsets were similar to the total NK cell population (Additional file 1: Figure S2F, G). We compared cytokine production in IA and IT patients in a IA:IT = 1:1 age-matched CHB cohort. Additional file 1: Figure S3 shows that the granzyme B, perforin, IFN-γ and TNF-α levels from NK cells were similar in the age-matched IA and IT group (*p* > 0.05). Collectively, these results demonstrated that NK cells of CHB patients exhibited an inhibitory phenotype, and the function of these cells was partially impaired. Notably, anti-viral cytokine secretion capacity and cytotoxicity were comparable between IA and IT patients when the effect of patient age was offset.

### Correlation of NK cell immunity with clinical virology characteristics

**Fig. 4:**
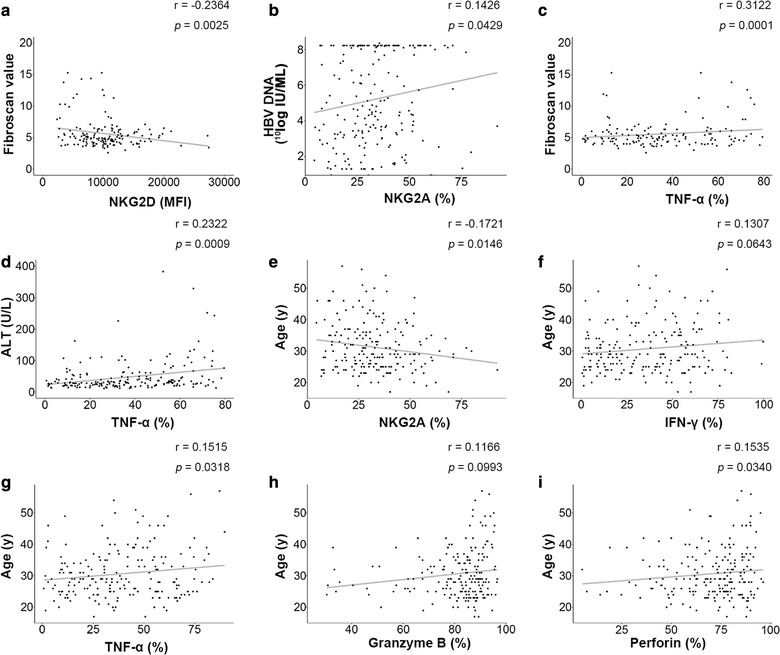
Correlation of NK cell immunity with clinical characteristics. Pearson correlation tests were performed between NK cell immunity markers (NKG2D, NKG2A, TNF-α, IFN-γ, granzyme B and perforin levels) and clinical parameters (Fibroscan values, HBV DNA, ALT levels and age).  Correlations between **a** NKG2D and Fibroscan value, **b** NKG2A and HBV DNA, **c** TNF-α and Fibroscan value, **d** TNF-α and ALT, **e** NKG2A and age, **f** IFN-γ and age, **g** TNF-α and age, **h** granzyme B and age, **i** perforin and age are shown

We analysed the correlation between NK cell phenotype/function and clinical parameters to clarify the relationship between NK cell phenotype/function with HBV replication and disease activity (Fig. 4). Our results demonstrated that NKG2D expression on NK cells negatively correlated with Fibroscan values in the CHB cohort (Fig. 4a). The percentage of NKG2A-positive NK cells positively correlated with serum HBV DNA level (Fig. 4b) and negatively correlated with patient age (Fig. 4e). The percentage of TNF-α-producing NK cells positively correlated with Fibroscan values and ALT levels (Fig. 4c, d, g). Four parameters that directly reflect NK cell function, including IFN-γ, TNF-α, perforin and granzyme B, positively correlated with age (Fig. 4f–i).

### Phenotype and function profiles of NK cells in CA and CAN patients

**Fig. 5:**
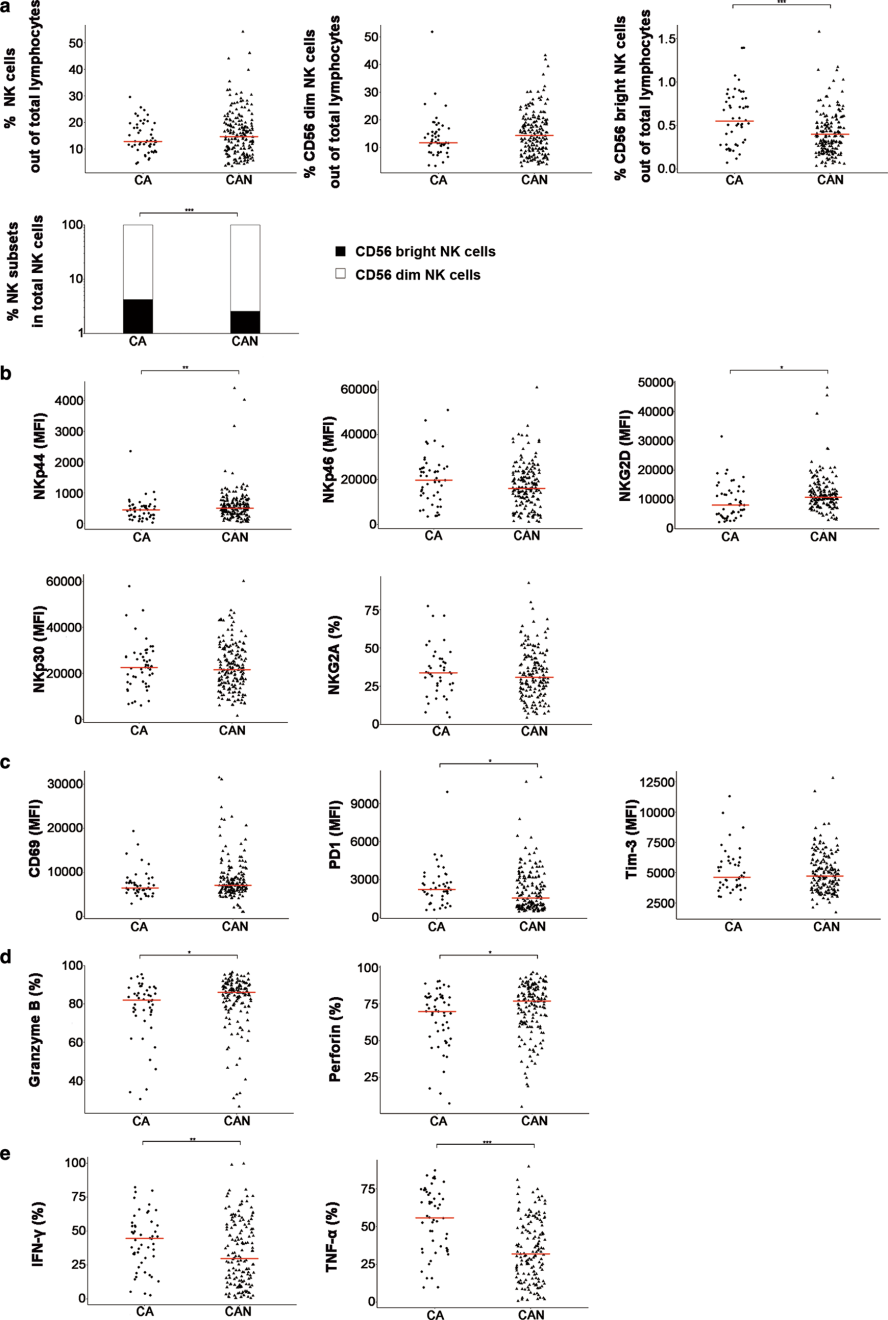
NK immunity in CA and CAN patients. **a** The frequency of total NK cells and their two subsets are shown for CA and CAN groups, and distribution of NK subsets is depicted as the frequency of CD56+CD3− NK cells. **b** MFI for NKp44, NKp46, NKG2D, and NKp30 on CD56+CD3− NK cells and the frequency for NKG2A^+^ cells within CD56+CD3− NK cells from CA and CAN groups. **c** MFI for CD69, PD1 and Tim-3 on CD56+CD3− NK cells from CA and CAN groups. **d**–**e** The frequency for granzyme B^+^, perforin^+^, IFN-γ^+^ and TNF-α^+^ cells within CD56+CD3− NK cells from CA and CAN groups. Horizontal bars represent the median. *CA* CHB patients strongly recommended to start antiviral therapy, and *CAN* CHB patients excluded from treatment based on the treatment guidelines

The current criteria for initiating antiviral therapy are based on ALT and HBV DNA levels. We stratified our CHB patient cohort into two groups: patients who were strongly recommended to start antiviral therapy (CA) and patients who not meet the criteria for treatment (CAN). Table 4 shows the clinical parameters of these two groups. We compared the percentage, phenotype and function of NK cells between these two groups. The percentage of CD56 bright NK cells compared to total lymphocytes in CAN patients decreased significantly compared to CA patients (Fig. 5a), but there was a distribution change towards more CD56 bright NK cells within the total NK cell population (Fig. 5a). Our results demonstrated that the expression of activating receptors NKG2D and NKp44 in the total NK cell population and their two subsets decreased in the CA group compared to the CAN group (Fig. 5b, Additional file 1: Figure S4A–E), but no differences were observed in the expression of the other NKRs. We investigated the phenotype of NK cells via detection of CD69, PD1 and Tim-3 expression. The expression of CD69 and Tim-3 expression did not differ between the two groups, but PD1 expression in NK cells in CA patients was significantly higher than CAN patients (Fig. 5c). Perforin and granzyme B production by the total NK cell population and their two subsets was higher in the CAN group than the CA group (Fig. 5d, Additional file 1: Figure S4I, J). In contrast, IFN-γ and TNF-α production in NK cells were higher in the CA group than the CAN group (Fig. 5e, Additional file 1: Figure S4K, L). These results demonstrated that the non-cytolytic functions of total NK cells and their two subsets were stronger in CA patients than CAN patients, which is consistent with the previous hypothesis that the immune response is more active in CA groups. However, NK cells in CAN patients exhibited stronger degranulation functions than CA patients.

**Table 4:** Clinical-virological characteristics of CA and CAN patients

Characteristics	CA (n = 54)	CAN (n = 183)	*p* value
Age, years	28 (25, 33.25)	29 (25, 36)	0.2847
Gender, n (%)			1.0000
Female	16 (29.6)	53 (29)	
Male	38 (70.4)	130 (71)	
Body mass index	20.56 (19.16, 22.73)	21.24 (19.42, 23.27)	0.4804
HBV DNA, log 10 IU/mL	7.913 (6.595, 8.23)	4.102 (2.717, 8.079)	0.0000
HBV genotype, n (%)			0.0043
B	29 (53.7)	91 (49.7)	
C	18 (33.3)	32 (17.5)	
Other	7 (13)	60 (32.8)	
HBeAg status, n (%)			0.0000
HBeAg positive	40 (74.1)	68 (37.2)	
HBeAg negative	14 (25.9)	114 (62.3	
Missing	0 (0)	1 (0.5)	
HBsAg, IU/mL	12,259.5 (2666.8, 50,107)	2600 (708, 22,849)	0.0002
Smoker, n (%)			0.5465
No	50 (92.6)	162 (88.5)	
Yes	4 (7.4)	21 (11.5)	
Vertical transmission, n (%)			1.0000
No	40 (74)	137 (74.9)	
Yes	7 (13)	24 (13.1)	
Missing	7 (13)	22 (112.0)	
ALT, U/L	101 (73, 161)	28 (20, 35.2)	0.0000
TBIL, mg/dL	15.3 (11.8, 20.8)	12.3 (9.6, 16.4)	0.0006
ALB, g/L	44.7 (41.9, 45.7)	46.4 (44.4, 48.1)	0.0008
FibroScan value, kPa	6.3 (5.2, 10.6)	4.8 (4.2, 5.6)	0.0000

Continuous variables were showed median (25, 75%)

## Discussion

The present study found that NK cell immunity was impaired in CHB patients. Notably, CHB patients in the IT and GZ phase exhibited partially preserved NK cell immunity and antiviral activity. We provided a comprehensive comparative analysis of NK cell immunity between CHB patients who met the stringent inclusion (CA) criteria for treatment and the CHB patients who were excluded from treatment based on the treatment guidelines (CAN).

Our data revealed NK cell immunity deficiencies in phenotype and function in CHB patients. NK cells of CHB patients exhibited an inhibitory phenotype with down-regulated expression of activating receptors NKp44 and NKp46 and up-regulated expression of exhaustion molecule Tim-3 compared to healthy controls, which indicates an exhaustion status and may explain the functional deficiency of NK cells in CHB patients. Previous studies demonstrated that the activating natural cytotoxicity receptors NKp44 and NKp46 participate in the induction of NK cell cytotoxicity and mediation of the production of pro-inflammatory cytokines by NK cells [22–24]. Deficiencies in these NKRs resulted in reduced NK cell activation and cytokine production, which was observed in animal models and HIV-infected patients [25, 26]. PD1 and Tim-3 are important exhaustion molecules that are expressed in different cell populations, including T cells and NK cells. Both of these factors are involved in the regulation of immune cells. A recent study demonstrated that PD-1-positive NK cells possessed impaired degranulation/cytotoxic activity and cytokine production and elicited poor proliferation in response to exogenous cytokines [27]. In vivo and in vitro experiments demonstrated that HBV increased Tim-3 expression on NK cells, and ex vivo blockade of Tim-3 with an anti-Tim-3 antibody in PBMCs or NK cells from CHB patients increased cytotoxicity and IFN-γ secretion [18]. We compared the function of NK cells between the CHB cohort and HC subjects and observed decreased cytokine production and preserved or elevated cytotoxicity. These functional changes indicate the functional dichotomy of NK cell functions in CHB patients, which was proposed by previous studies [20, 21]. The inhibitory phenotype and decreased cytokine production of NK cells in CHB patients provide clues of the effect of chronic HBV infection on NK cell status.

We did not find decreased NKG2A in total NK cells or its two subsets in CHB patients compared to healthy controls, but some variance in NKG2A levels in the CD56 bright subset was observed between different CHB stages. NKG2A is an inhibitory receptor that is expressed on NK cells. This receptor was increased on NK cells from CHB patients and decreased with HBV DNA levels during anti-virus therapy [14]. We also found that NKG2A levels on the CD56 bright subset positively correlated with HBsAg, HBV DNA and HBeAg levels (data not shown). The classification criteria of different stages primarily consider HBV DNA level, HBeAg status and ALT level. Therefore, the variance in NKG2A expression on CD56 bright NK cells may be attributed to virological discrepancies in different CHB patients.

No other NK cell phenotypical molecules correlated with HBsAg, HBV DNA or HBeAg levels. These molecules may play a role in the interface of NK cells with other cells (e.g., NK-DC crosstalk) rather than directly interactions with the virus [28]. Most stratified CHB subgroups possessed a similar phenotype, but the functional differences were remarkable. Patients in the IA phase were traditionally thought to be immune active because of their high ALT levels. Our data demonstrated that cytokine production in IA patients was the highest of the different CHB stages. This higher cytokine secretion ability in the IA group consisted of a shift to more CD56^bright^ NK cells, which specializes in cytokine release [29]. However, the cytotoxicity function of these cells were comparable with the IC and IT patients. Notably, the GZ patients possessed a stronger cytotoxicity function than IA patients.

The current dogma characterises CHB patients in the IT phase by high serum HBV DNA levels and normal ALT levels, and these patients are considered to have inactive immunity and are not treatment candidates. However, our data do not support this dogma. We found that the IT patients possessed a comparable cytotoxicity function as IA patients with decreased cytokine production ability. The preservation of NK cell cytolytic capacity is an important aspect of NK cell antiviral function, which is needed for the elimination of HBV cccDNA and the resolution of chronic infection [30, 31]. Figure 3c shows that the cytokine secretion capacity of IT patients was lower than IA patients. In contrast, recent studies found that some “immunotolerant” children retained IFN-γ secretion capability compared to HC children [28]. Our data found a positive correlation between age and anti-viral cytokines and the cytotoxicity function of total NK cells and its subsets, which indicates that age may be a factor influencing NK cell immunity. We further compared the NK cell immunity in two groups of age-matched IA and IT patients to exclude the interference of age on NK immunity (Additional file 1: Figure S3). These results indicated that similarly aged IA and IT patients may possess similar cytokine secretion capabilities and cytotoxicity function. However, the age-matched cohort we used only included a small group of patients, and the results should not be over-interpreted. More research is needed to identify the impact of CHB patient age on NK cell immunity. Our data did not find a correlation between IFN-γ secretion and cytolytic capacity or ALT level. Previous studies using an HBV transgenic mouse model demonstrated that HBV-specific T cells controlled HBV in transgenic mice in a non-cytopathic fashion via antiviral cytokines in addition to directly killing HBV-replicating hepatocytes, which indicates that elevated ALT is not necessary for immune response [32–34]. Taken together, our results and recent findings from other researchers argue against a complete tolerance of an NK cell immune response in IT patients. The IT phase, as defined by ALT, HBV DNA and HBeAg status, did not reflect the veritable NK cell immune status.

We provide a new insight into GZ patients who did not meet the IA, IC and IT stage criteria [3]. Few studies focus on these patients, but they are a large group of CHB patients who comprised approximately one-third of our CHB cohort. Our data demonstrated that the cytolytic capacity of NK cells in this group was higher than the HC group and IA patients, and some GZ patients retained the ability to secrete antiviral cytokines similarly to IA patients. These results indicate that the anti-viral functions are preserved in GZ patients, and they may respond to anti-virus therapy similarly to IA patients.

Previous studies demonstrated that the rate of fibrosis progression increased when the CHB patients who remained in the IT phase progressed into the immune clearance phase [35], and a sizable fraction of the GZ patients (9%) can progress into cirrhosis [3]. Significant histological evidence of fibrosis is found in liver biopsies in a fair proportion of patients with chronic HBV infection and normal ALT levels [32–34]. Therefore, the clinical stage of CHB patients based on HBV DNA and ALT levels does not sufficiently reflect the veritable immune status, and more precise methods are needed. The traditional opinion that an immune tolerant status with normal ALT level restricts anti-viral therapy should be reconsidered. Earlier initiation of treatment to patients, including IT phase and GZ groups, may induce HBV immune control and avoid a poor prognosis.

The current treatment criteria were established based on 3 clinical parameters: ALT, HBV DNA and HBeAg status. We found that these 3 clinical parameters did not sufficiently reflect the veritable immune status in patients with different CHB stages. Therefore, we further evaluated whether current treatment criteria provide sufficient information for treatment and compared NK cell immunity between patients who were strongly recommended to start antiviral therapy (CA) and patients who did not meet the criteria for treatment (CAN) patients. Patients in the CAN group expressed more activating receptors and less exhaustion molecules and secreted higher levels of perforin and granzyme B compared to patients in the CA group. The phenotype and degranulation function of NK cells in the CAN group indicated that the innate immune response in these patients was partially preserved. The average secretion level of INF-γ and TNF-α in the CAN group was lower than the CA group, but the cytokine secretion ability of the CAN group was heterogeneous. These results suggest that the CAN group consisted of patients with heterogeneous immune states, and some patients may possess an immune response that is comparable to CA patients without clinically active liver disease. PD1 was increased in T cells from CHB patients compared to healthy controls, which suggests that PD1 plays a role in immune dysregulation [2, 19]. Our study found no difference between CHB and HC groups, but PD1 expression on NK cells in the CAN group were lower than the CA group, which suggests that NK cell status in the CAN group was less exhausted. This finding warrants further investigation to examine the impact of increased PD1 expression on NK cell function. Current anti-viral agents for CHB are potent and highly efficacious with minimal side effects and a high barrier to resistance. The radical use of anti-viral agents may be more beneficial because of the reduction in the likelihood of poor clinical outcomes with less risk and may boost the immune system to control HBV infection in these situations, as shown in ex vivo experiments [18]. These patients may benefit from early antiviral therapy to prevent terminal liver damage. Future studies are required to develop methods to select these immune-active patients from the CAN group to initiate anti-viral treatment.

There were several limitations in our study. First, our study was a cross-sectional study, and all of our samples were obtained at a single time point. Follow-up data were not available in our study. Longitudinal research will allow for the observation of dynamic changes in NK cell immune status and its impact on disease fluctuations. Our future studies will focus on this aspect. Second, we examined peripheral NK cells in this study, but there are some differences between intrahepatic NK cells and their peripheral counterparts. We will further investigate intrahepatic NK immunity in the future.

## Conclusions

We revealed the comprehensive characteristics of NK cells in CHB patients during different clinical stages of CHB infection and found that the immune function of NK cells was partially preserved in the IT and GZ phases. We provide the first evidence that CHB patients who were excluded from treatment based on the current treatment guidelines did not show completely worse immunity than patients who were eligible for treatment. These results suggest that the immune classification of CHB patients based on the HBV DNA and ALT levels does not accurately reflect the veritable immune status and that some patients in the IT phase and “grey zone” should be considered for earlier initiation of anti-viral therapy.

## Additional file

**Additional file 1.** Supplementary Figures 1–4.

## Abbreviations

HBV: hepatitis B virus
CHB: chronic hepatitis B
NK: natural killer cells
DC: dendritic cells
IFN: interferon
TNF: tumour necrosis factor
IT: immune tolerant phase
IA: immune active phase
IC: inactive CHB phase
GZ: grey zone
ALT: alanine aminotransferase
ULN: upper limit of normal
CA: patients who are recommended to initiate antiviral therapy
CAN: patients who are excluded from treatment according to the guidelines
HC: healthy controls
BMI: body mass index
HBeAg: hepatitis B e antigen
HBsAg: HBV surface antigen
ALB: albumin
TBIL: total bilirubin
PBMCs: peripheral blood mononuclear cells
NKR: NK cell receptor
PD1: programmed death-1
Tim-3: T cell immunoglobulin- and mucin-domain-containing molecule-3
