# Supplementary material for: Veritable antiviral capacity of natural killer cells in chronic HBV infection: an argument for an earlier anti-virus treatment
Source: J Transl Med. 2017 Oct 31;15:220. doi: 10.1186/s12967-017-1318-1 (PMC5663047; doi:10.1186/s12967-017-1318-1)
Supplement: Supplementary file 1 — Additional file 1. Supplementary Figures 1–4. [file 12967_2017_1318_MOESM1_ESM.docx]

**Additional Figures**


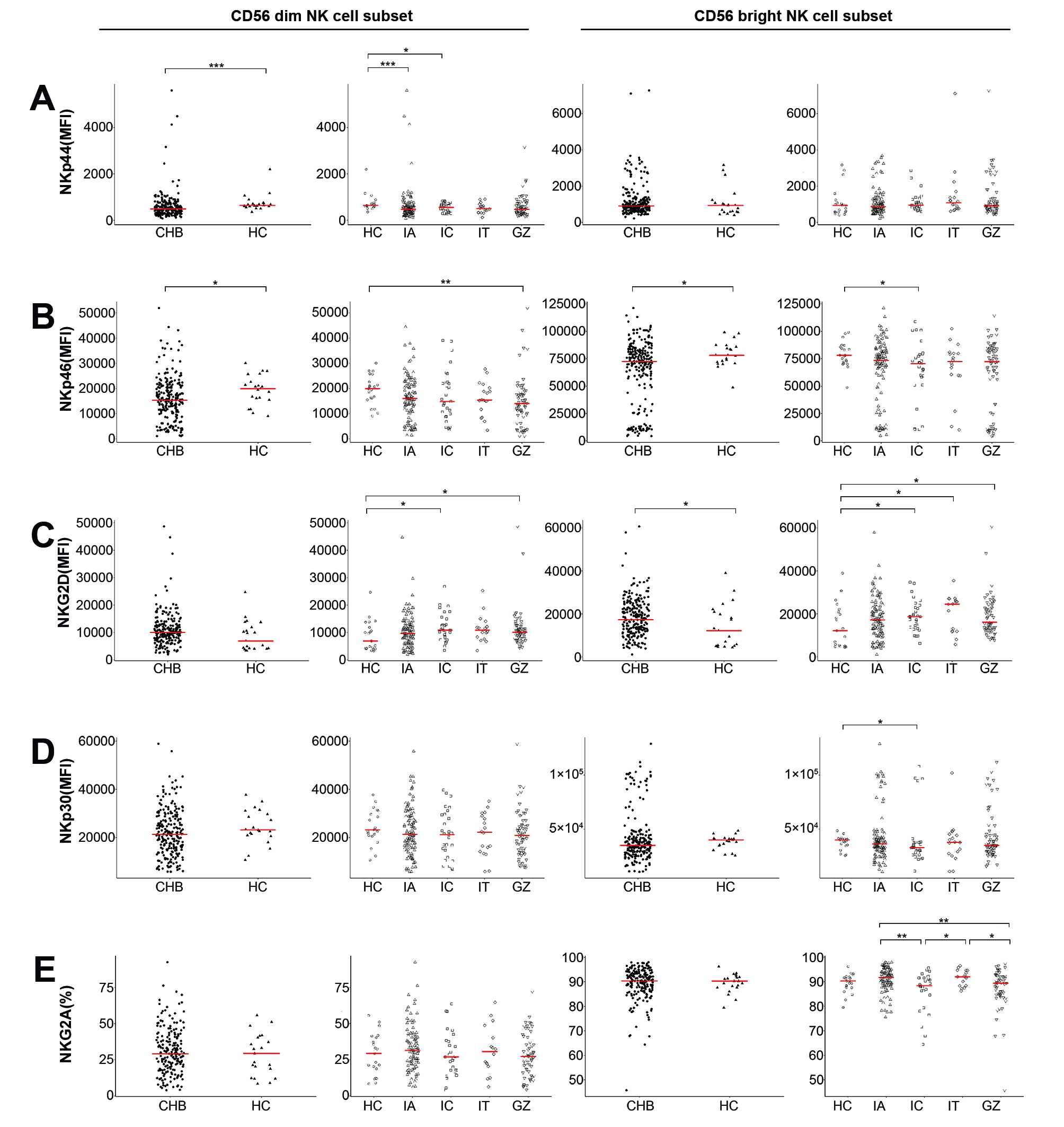


**Figure S1 Receptor expression characteristics of NK subsets in treatment naïve CHB patients**

Summary of the expression (MFI) of NKp44 (A), NKp46 (B), NKG2D (C), and NKp30 (D) the percentage of NKG2A^+^(E) in NK^dim^ and NK^bright^ cells in the CHB patients and patients in different CHB phases.

and horizontal bars represent the median. Abbreviations: CHB, chronic hepatitis B; HC, healthy control; IA, immune active; IT, immune tolerance; IC, inactive CHB; and GZ, grey zone.


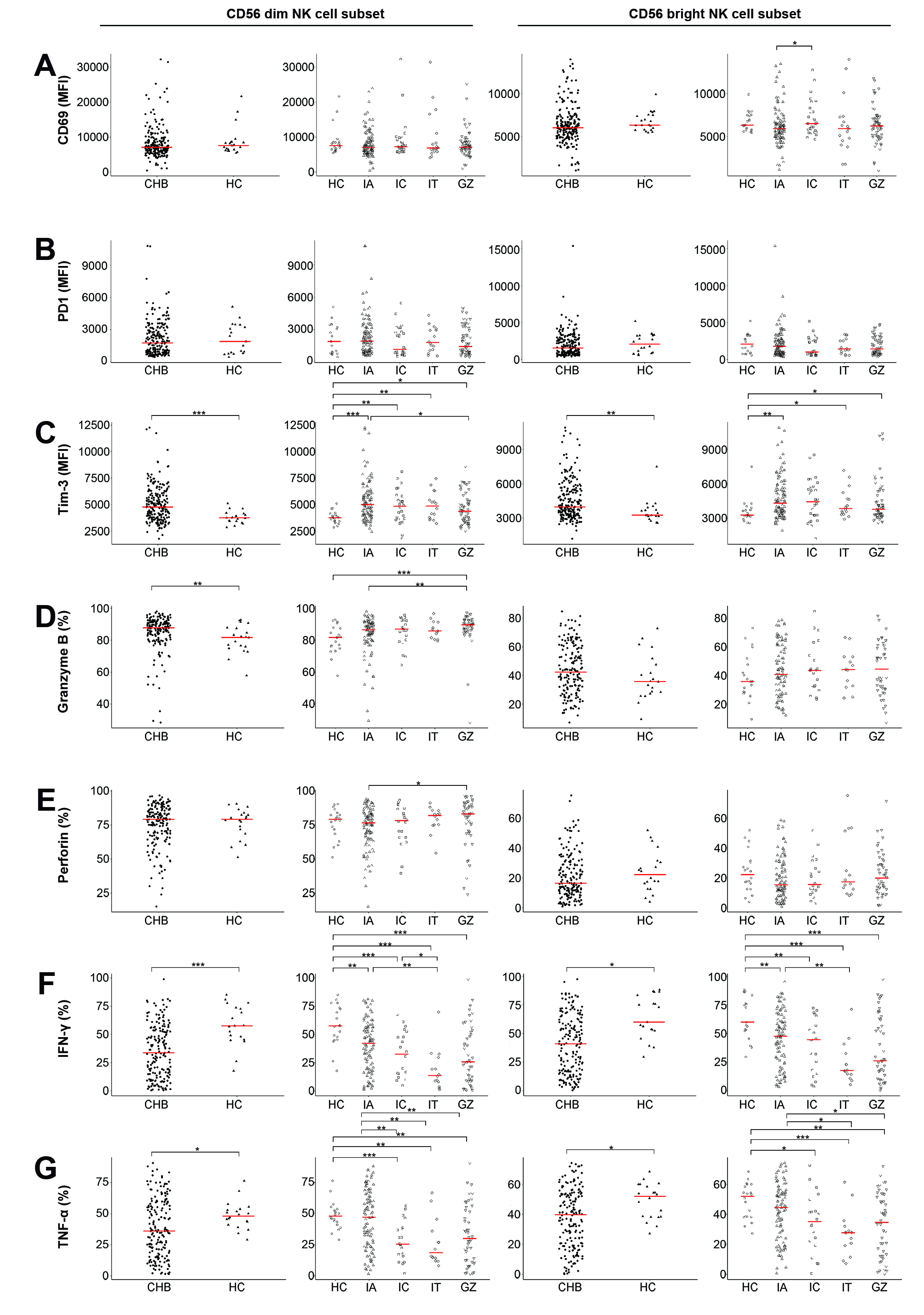


**Figure S2 Analysis of function profiles of NK subsets in CHB patients**

(A-C) The expression (MFI) of CD69 (A), PD1(B) and Tim-3 (C) in NK^dim^ and NK^bright^ cells

(D-G) The frequency of granzyme B^+^ (D), perforin^+^ (E), IFN-γ^+^ (F) and TNF-α^+^ (G) NK^dim^ and NK^bright^ cells in the total CHB group and the different CHB clinical phase groups plus the HC group.

and horizontal bars represent the median. Abbreviations: HBV, hepatitis B; CHB, chronic hepatitis B; HC, healthy control; IA, immune active; IT, immune tolerance; IC, inactive CHB; and GZ, grey zone.


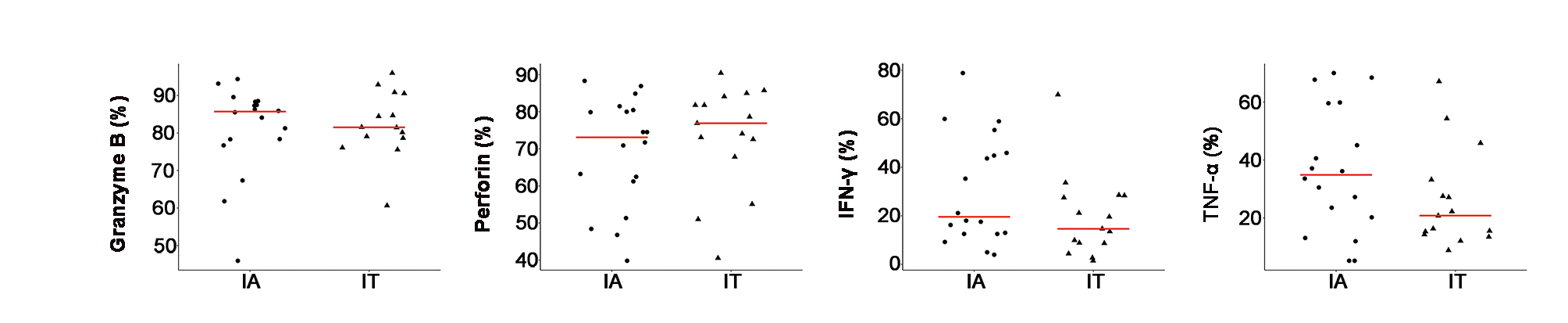


**Figure S3 Function profiles of NK cell in IA-IT age-matched cohort**

Dot plots comparing the frequency of granzyme B^+^ , perforin^+^ , IFN-γ^+^ and TNF-α^+^ NK cells in total NK cells in IA and IT groups.The IA-IT age-matched cohort was composed of all 18 IT patients in the whole CHB cohort and 18 IA patients who are age-matched to those IT patients.


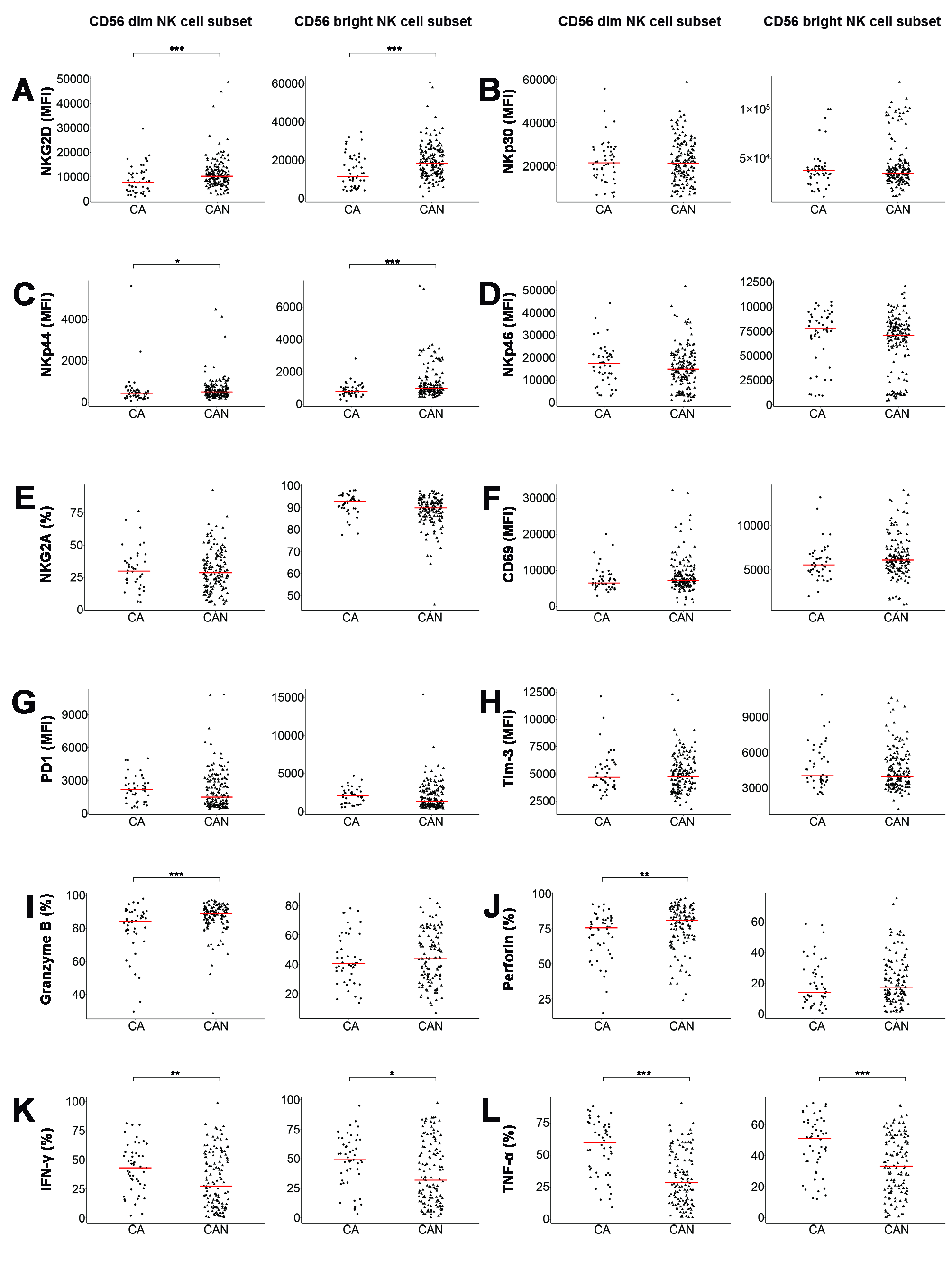


**Figure S4 Phenotype and function of NK subsets in CA and CAN patients**

(A-E) Comparison of the expression (MFI) of NKp44, NKp46, NKG2D, NKp30 and the percentage of NKG2A^+^ cells among NK^dim^ and NK^bright^ subsets in CA and CAN groups.

(F-H) The expression (MFI) of CD69, PD1 and Tim-3 in NK^dim^ and NK^bright^ subsets in CA and CAN groups.

(I-L) The production of granzyme B, perforin, IFN-γand TNF-αby NK^dim^ and NK^bright^ subsets in the CA and CAN groups.

and horizontal bars represent the median. Abbreviations: CA, CHB patients strongly recommended to start antiviral therapy; and CAN, CHB patients excluded from treatment based on the treatment guidelines.
